# Deciphering unique and shared interactions between the human gut microbiota and oral antidiabetic drugs

**DOI:** 10.1002/imt2.179

**Published:** 2024-03-13

**Authors:** Huahui Ren, Zhun Shi, Fangming Yang, Shujie Wang, Fengyi Yuan, Tingting Li, Min Li, Jiahui Zhu, Junhua Li, Kui Wu, Yifei Zhang, Guang Ning, Karsten Kristiansen, Weiqing Wang, Yanyun Gu, Huanzi Zhong

**Affiliations:** ^1^ BGI Research Shenzhen China; ^2^ Department of Biology Laboratory of Genomics and Molecular Biomedicine University of Copenhagen Copenhagen Denmark; ^3^ Department of Endocrine and Metabolic Diseases Shanghai Institute of Endocrine and Metabolic Diseases, Ruijin Hospital Shanghai Jiao Tong University School of Medicine Shanghai China; ^4^ Shanghai National Clinical Research Center for Metabolic Diseases Key Laboratory for Endocrine and Metabolic Diseases of the National Health Commission of the PR China, Shanghai National Center for Translational Medicine, Ruijin Hospital Shanghai Jiao Tong University School of Medicine Shanghai China; ^5^ Department of Endocrinology and Metabolism Shenzhen People's Hospital Shenzhen China; ^6^ Shenzhen Key Laboratory of Unknown Pathogen Identification, BGI Research Shenzhen China; ^7^ Guangdong Provincial Key Laboratory of Human Disease Genomics, Shenzhen Key Laboratory of Genomics, BGI Research Shenzhen China

## Abstract

The administration of oral antidiabetic drugs (OADs) to patients with type 2 diabetes elicits distinct and shared changes in the gut microbiota, with acarbose and berberine exhibiting greater impacts on the gut microbiota than metformin, vildagliptin, and glipizide. The baseline gut microbiota strongly associates with treatment responses of OADs.

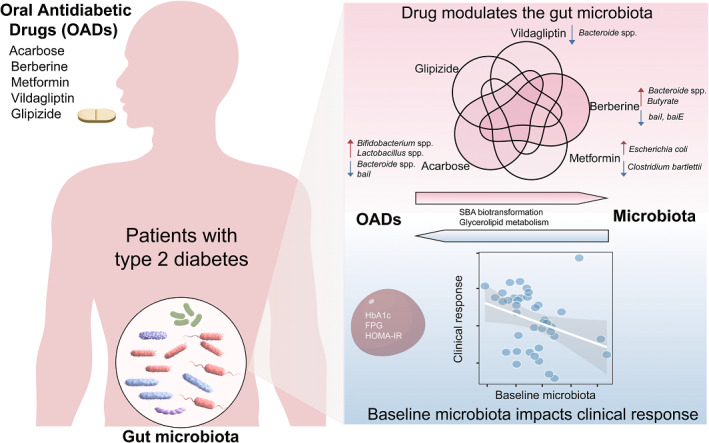

##  

Type 2 diabetes (T2D) is a chronic metabolic disorder characterized by hyperglycemia and increased insulin resistance, and is closely associated with gut microbial dysbiosis [1]. Oral antidiabetic drugs (OADs), such as metformin, sulfonylureas, alpha‐glucosidase inhibitors (AGIs), and dipeptidyl‐peptidase‐4 (DPP‐4) inhibitors are commonly prescribed for glycemic control in T2D patients, yet the effectiveness of such treatment displays considerable variations across individuals.

A number of studies have demonstrated that various OADs significantly alter the gut microbiota, which in turn have profound effects on host metabolism underlying in part the clinical benefits in individuals with T2D [2, 3, 4, 5, 6]. The administration of metformin significantly increased the relative abundance of *Akkermansia muciniphila* [5]. Acarbose, an AGI, was found to dramatically alter the gut microbial composition by elevating *Bifidobacterium* and depleting *Bacteroides* spp. [4, 7]. Our recent study demonstrated that a 12‐week berberine treatment significantly reduced the abundance of *Ruminococcus bromii*, a secondary bile acid (SBA)‐producer, and plasma levels of deoxycholic acid (DCA), associated with improved glucose homeostasis [6]. Additionally, two studies, including ours, have revealed associations between the baseline gut microbiota and therapeutic outcomes of DPP‐4 inhibitors [7, 8].

However, considerable disparities persist in the reported gut microbial alterations in response to different OADs, possibly due to the variations in ethnicity, sample size, and metagenomic methodology across studies. A comprehensive evaluation using consistent pipelines is clearly warranted to understand bidirectional interactions between OADs and the gut microbiota, as well as their potential impacts on drug efficacy.

## RESULTS AND DISCUSSION

### Study characteristics and clinical outcomes of OADs in T2D patients

This study encompassed 470 T2D patients from six clinical trials [4, 5, 6, 7, 9] (Table S1 and Figure S1). Five publicly available studies were included, and an open‐labeled, single‐arm clinical trial (NCT04426422) was conducted in the present study, including 47 participants who completed a 3‐month metformin treatment. Except for the study of Zhao et al. [9], the remaining studies enrolled only newly‐diagnosed‐T2D (ND‐T2D) patients, providing baseline and posttreatment glycemic measures (Table 1). The intervention durations varied (84–168 days) across different trials. In addition, participants in the study of Wu et al. [5] were from Spain, while the rest were Chinese. Overall, Chinese participants had a mean age of 51.84 years, with 55.3%–61.7% being men, a mean body mass index (BMI) of 26.2 kg/m^2^, and a baseline hemoglobin A1C (HbA1c) above 7.5%. By contrast, the Spanish participants had a higher BMI (36.5 kg/m^2^) and a lower baseline HbA1c (6.67%) than the Chinese participants (Table 1). All OADs led to effective reductions in HbA1c, fasting glucose (FPG), and 2‐h postprandial glucose (Figure S2).

**Table 1:** Details of oral antidiabetic drugs (OADs) related metagenomic data sets included in this study.

Data set	Disease	Drug (participant/sample)	Drug dosage/duration	Sampling (days)	Age (years)	Sex (male/female)	BMI (kg/m^2^, Pre)	HbA1c (%, pretreatment)	HbA1c (%, posttreatment)	Location	Accession number
Gu et al. [4]	ND‐T2D	Acarbose (51/102)	100 mg tid p.o. (minimum)/3‐month	90	52.96 ± 0.95	17/34	26.32 ± 0.45	7.53 ± 0.11	6.39 ± 0.07^a^	China, Multicenter	PRJEB12124
		Glipizide (43/86)	5 mg tid p.o./3‐month	90	53.96 ± 1.03	24/19	26.01 ± 0.52	7.67 ± 0.14	6.32 ± 0.10^a^		
Wu et al. [5]	ND‐T2D	Metformin (22/65)	1,700 mg tid p.o./4‐month	60, 120	52.6 ± 2.0	8/14	36.54 ± 1.44	6.67 ± 0.11	5.97 ± 0.08^a^	Spain	PRJNA361402
Zhang et al. [6]	ND‐T2D	Berberine (85/170)	600 mg bid p.o./12‐week	84	52 ± 1.17	52/33	25.86 ± 0.37	7.68 ± 0.08	6.68 ± 0.07^a^	China, Multicenter	PRJNA643353
		Placebo (96/192)	600 mg bid p.o./12‐week	84	52.23 ± 0.99	56/40	26.32 ± 0.35	7.83 ± 0.08	7.22 ± 0.10^a^		
Zhang et al. [7]	ND‐T2D	Acarbose (42/84)	100 mg tid p.o./24‐week	168	52.19 ± 1.48	27/15	26.87 ± 0.27	7.82 ± 0.09	6.40 ± 0.09^a^	China, Bei Jing	PRJNA826552
		Vildagliptin (40/80)	50 mg bid p.o/24‐week	168	51.17 ± 1.4	19/21	27.11 ± 0.28	7.78 ± 0.10	6.36 ± 0.10^a^		
Ren et al. (this study)	ND‐T2D	Metformin (47/94)	1500 mg tid p.o./3‐month	90	47.83 ± 1.38	26/21	25.26 ± 0.42	8.20 ± 0.22	6.36 ± 0.11^a^	China, Shen Zhen	CNP0004692
Zhao et al. [9]	T2D	Acarbose + U (16/64)	100 mg tid p.o./12‐week	28, 56, 84	59.7	7/9	NA	8.31 ± 0.38	7.01 ± 0.27^a^	China, Shang Hai	PRJEB14155
		Acarbose + W (27/108)	100 mg tid p.o./12‐week	28, 56, 84	58.4	11/16	NA	8.27 ± 0.27	6.36 ± 0.11^a^		

*Note*: Continuous data are presented as mean ± sem (standard error of mean).

Abbreviations: Acarbose + U, Acarbose + usual care; Acarbose + W, Acarbose + WTP (whole grains, traditional Chinese medicinal foods, and prebiotics); BMI, body mass index; HbA1c, hemoglobin A1C; ND‐T2D, newly diagnosed‐T2D; T2D, type 2 diabetes.

^a^ HbA1c reported significantly decreased in the study (*p* < 0.05).

### Impacts of OADs on the human gut microbiota

A total of 1, 045 metagenomic datasets were collected and processed using identical pipelines, yielding an average of 98.4 million clean reads per sample (Table S2). Considering the substantial baseline variations in microbial composition among studies, particularly between Spanish and Chinese participants, all metagenome‐based analyses were performed within each study (Figure S3).

**Figure 1:**
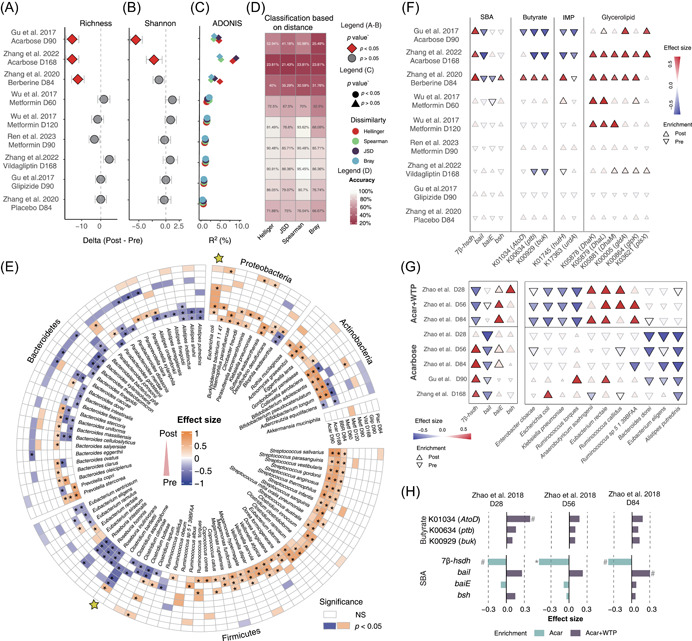
Overview of microbial compositional alterations induced by five oral antidiabetic drugs in eight intervention groups. (A, B) Forest plots showing changes (delta) in measures of alpha diversity, including richness (A) and Shannon index (B), between pre‐ and post‐treatment samples. Wilcoxon signed‐rank test. Circles indicate non‐significant changes (*p* > 0.05), and diamonds indicate significant changes (*p* < 0.05). (C) PERMANOVA indicating the extent of within‐group differences in gut microbial composition between pre‐ and post‐treatment samples, quantified by the variation explained (*R* ^2^). Four dissimilarity metrics were employed: Hellinger (red); Spearman (Spearman's rank coefficient, green); Jensen–Shannon divergence (JSD, black), and Bray–Curtis distance (blue). (D) Heatmap displaying individual classification/assignment accuracy based on the four dissimilarities measures from panel (C). (E) Heatmap showing species exhibiting significant changes in relative abundance comparing pre‐ and post‐treatment in the eight treatment groups. Species exhibiting changes in abundance in at least one single trial are presented and ranked by phylum: Bacteroidetes, Firmicutes, Proteobacteria, Actinobacteria, and Verrucomicrobia. Colors represent effect size from the Wilcoxon signed‐rank test: orange for species increased in abundance, blue for species decreased in abundance, and white for species exhibiting no significant changes in abundance (NS) after treatment. *Benjamini–Hochberg (BH)‐adjusted *p* < 0.05. (F) Dot plot showing the alterations in relative abundances of gut microbial genes involved in secondary bile acid biotransformation, butyrate, imidazole propionate (ImP), and glycerolipid metabolism. Blue triangles indicate decreased relative abundances of genes and species, and orange triangles indicate increased relative abundances using the Wilcoxon signed‐rank test. A BH‐adjusted *p* < 0.05 was considered statistically significant. The gut microbial genes include *7β‐hsdh*, *baiI*, *baiE*, *bsh*, K01034 (*AtoD*), K00634 (*ptb*), K00929 (*buk*), K01745 (*hutH*), K17363 (*urdA*), K05878 (*DhaK*), K05979 (*DhaL*), K05881 (*DhaM*), K00005 (*gldA*), K00864 (*glpK*), and K03621 (*plsX*). (G) Dot plot showing changes in the relative abundances of microbial species or genes induced by acarbose alone and acarbose combined with a high‐fiber diet (Acar+WTP). Blue triangles indicate decreased relative abundances of genes and species, and orange triangles indicate increased relative abundances using the Wilcoxon signed‐rank test. A BH‐adjusted *p* < 0.05 was considered statistically significant. (H) Bar plot showing the differences in relative abundances of gut microbial genes involved in SBA and butyrate production. Colored bars indicate effect sizes estimated from comparisons between post‐treatment samples between acarbose alone and Acar+WTP using Wilcoxon rank sum tests. Light green indicates enrichment in the Acar group and dark gray indicates enrichment in Acar+WTP. Dashed line indicates an absolute value of effect size at 0.3. *BH‐adjusted *p* < 0.05, ^#^ *p* < 0.05. Acar, Acarbose; SBA, secondary bile acid; WTP, whole grains, traditional Chinese medicinal foods, and prebiotics.

We initially evaluated the effects of OAD treatment on the gut microbiota of ND‐T2D patients, including a placebo control group from the study of Zhang et al. [6]. Notably, both acarbose and berberine treatments significantly reduced species‐level richness and Shannon index, and altered community composition (Bray–Curtis, Hellinger, Spearman dissimilarity, and Jensen–Shannon divergence) (Figures 1A–C and S4A–B). By contrast, such changes were not observed following the administration of metformin, vildagliptin, glipizide, or placebo (Figures 1A–C and S4A–B). To evaluate the impact of treatment, we employed a distance matrix‐based approach testing if pre‐ and posttreatment samples clustered together. At the individual level, more than half of the post‐treatment samples from the acarbose and berberine groups could not be accurately classified/assigned according to individuals, but for the other groups, individual classification/assignment was predicted with high accuracy (>70%) (Figure 1D), highlighting the profound impacts of acarbose and berberine in altering the individual‐specific microbial fingerprint [10].

At the species level, unique and shared alteration patterns were characterized among different trials (Figure 1E and Table S3). Both acarbose and berberine significantly increased the relative abundances of multiple Firmicutes members (Figures 1E and S4C). Conversely, a reduction in relative abundances of Bacteroidetes members was observed in response to acarbose and vildagliptin treatments, including *Bacteroides xylanisolvens, Bacteroides thetaiotaomicron, Bacteroides cellulosilyticus*, and *Alistipes shahii* (Figures 1E and S4C). Additionally, berberine led to a significant increase in *Bacteroides* spp. and a reduction in *Bifidobacterium* spp., exhibiting opposite changes compared with acarbose (Figure 1E). Alterations in the abundance of two gut microbial species in response to metformin treatment, that is, an increase in the relative abundance of *Escherichia coli* and a decrease in the relative abundance of *Clostridium bartlettii* [2, 5, 11], were also observed in the berberine group (Figures 1E and S4D). Except for metformin, *A. muciniphila* showed no significant changes in abundance in other trials or the placebo group (Figure 1E). We further evaluated the influence of OADs on species–species network, revealing that most species displayed positive correlations, and both acarbose and berberine strongly enhanced species interactions, reflected in increased edges numbers, density, connectivity, clustering coefficient, and decreased modularity (Figure S5 and Table S4–S5).

At the functional level, both acarbose and berberine significantly increased the relative abundances of the gene encoding 7β hydroxysteroid dehydrogenase (*7β‐hsdh*, involved in the production of ursodeoxycholic acid, UDCA), while concurrently reducing the relative abundances of genes involved in SBA bitransformation, namely, the 7ɑ‐dehydratase (*baiE*) and/or 7β‐dehydratase (*baiI*) (Figure 1F and Table S6). Similar trends were observed in the metformin study of Wu et al. [5] (*p* < 0.05 for *baiE*, Figure 1F). Consistently, we observed elevated levels of UDCA and reduced levels of DCA in response to acarbose or berberine (Figure S6A–C), with species associated with *baiE*/*baiI* largely overlapping with those reduced by the two treatments (Figure S6D). Additionally, only berberine led to a notable increase in the relative abundances of the bile salt hydrolase (*bsh*) gene. Except for glipizide, all other OADs increased the abundances of genes involved in glycerolipid metabolism (Figure 1F and Table S7). Consistently, we identified significant longitudinal associations between changes in microbial features and host glucose levels, including positive associations between changes in the abundance of *B. xylanisolvens* and HbA1c following administration of acarbose and vildagliptin, positive associations between changes in the abundance of *C. bartlettii* and HbA1c in individuals treated with metformin and berberine, and negative associations between changes in *7β‐hsdh* and HbA1c in acarbose and berberine treated individuals (Figure S7, generalized estimating equation).

Subsequently, we integrated metagenomic data from Zhao et al. [9], Gu et al. [4], and Zhang et al. [7] to examine overall microbial alterations induced by acarbose alone and acarbose combined with a high‐fiber diet (Acar+WTP). In line with the original observations [9], Acar+WTP, in comparison to acarbose alone, modified the microbial composition by increasing the relative abundances of butyrate producers (i.e., *Anaerobutyricum hallii*, *Eubacterium rectale*, and *Ruminococcus callidus*) and reducing prevalent *Enterobacteriaceae* members (Figures 1G and S8, and Table S8). Our study further demonstrated no significant reductions in relative abundances of SBA biotransformation genes in the Acar+WTP group (Figure 1G and Table S9). This might be partially attributed to the fact that several Acar+WTP responsive species, such as *E. rectale*, *Eubacterium eligens*, and *A. putredinis*, have been identified as major SBA producers in the human gut [12]. Acar+WTP even led to a significant reduction in the relative abundance of the *7β‐hsdh* gene, exhibiting opposite patterns to the acarbose alone groups (Figure 1G and Table S9). Consequently, post‐treatment samples in the Acar+WTP group exhibited higher abundances of the butyrate producing gene (K01034) and *baiI*, and lower relative abundances of the *7β‐hsdh* gene compared with the acarbose‐alone groups (Figure 1H).

By applying consistent pipelines, we demonstrated shared and unique microbial alterations in response to different OADs, highlighting how different drugs impact on the gut microbiota of participants with T2D. While acarbose alone induced similar microbial changes over 28–168 days, a study by Sun et al. found a significant decrease in *Bacteroides* and *bsh* gene abundances after a 3‐day metformin intervention [13], not observed after 2–4 months of metformin treatments. Importantly, the gut itself is now recognized as another site of metformin action, impacting mitochondrial respiration, lactate production, and the bile acid pool within the intestine [14]. Further clinical studies with closely spaced sampling time points are needed to uncover the dynamic microbial changes and host‐microbiota interplays in response to metformin.

Our study unveiled the dual effects of the Acar+WTP combination in modulating the gut microbial composition and functional potentials in Chinese T2D patients by reversing the increased abundance of the *7β‐hsdh* gene and alleviating the reduction in the *baiI* gene induced by acarbose alone. The short‐term beneficial effects from fiber fermentation possibly outweighed the negative effects of SBAs production, leading to an overall improved therapeutic outcome in the combination group [9]. Notably, recent mouse studies have implicated an inulin‐fiber containing diet in triggering a cholic acid‐FXR‐dependent intestinal inflammation [15], and hepatocellular carcinoma [16] in a gut microbiota‐dependent manner. Specifically, the prolonged exposure to microbiota‐derived butyrate could increase hepatocyte growth and promote liver fibrosis [16]. Acarbose, with its potent alpha‐glucosidase inhibition activity, may enhance microbial utilization of fibers by facilitating the passage of undigested fiber to the colon, leading to production of fiber‐derived butyrate. However, the long‐term effects of combining AGIs with dietary fibers on metabolic health in T2D patients warrant further investigation.

### Baseline microbial features associate with treatment responses of OADs

**Figure 2:**
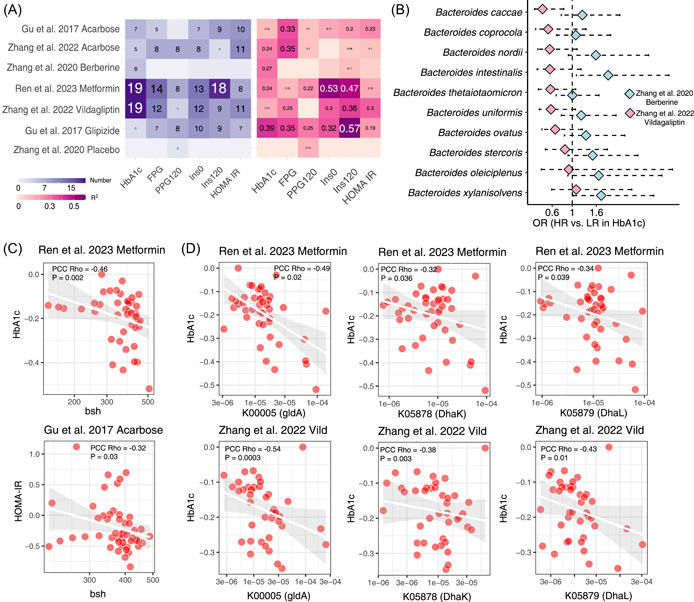
Links between baseline gut microbiota and treatment responses induced by different OADs. (A) Left heatmap displaying the number of microbial features that showed significant associations with treatment responses in each group. Significant associations were defined using an absolute correlation coefficient >0.3 and *p* < 0.05 (partial Spearman's correlation). Right Heatmap showing the variance of OAD‐induced changes in each clinical parameter explained by microbial features, evaluated by the *R* ^2^ from Elastic‐net regression models. (B) Forest plot showing the comparisons of relative abundances of *Bacteroides* spp. between the low responders and high responders (defined by the median of percentage change of HbA1c) to vildagliptin and berberine treatment. Odds ratios (OR) and 95% CI were estimated by logistic regression model. (C) Scatter plots showing the associations between the baseline relative abundances of *bsh* and treatment responses in metformin and acarbose groups. (D) Scatter plots showing the associations between the baseline relative abundances of genes involved in glycerolipid metabolism (K00005, K05878, and K05879) and treatment responses in metformin and vildagliptin groups. CI, confidence interval; FPG, fasting glucose; HbA1c, hemoglobin A1C; HOMA‐IR, Homeostatic Model Assessment of insulin resistance; HR, high responders; LR, low responders; OAD, oral antidiabetic drug; PPG, postprandial glucose.

We examined how and to what extent the baseline gut microbiota may predict treatment responses across the different trials using Elastic‐net regression models. Baseline microbial features explained 12%–39% of the response variance in HbA1c, 12%–35% of the variance in FPG, and 6%–30% of the variance in HOMA‐IR across OADs groups (Figure 2A and Tables S10–S11). Notably, higher baseline abundances of *Bacteroides* spp. tended to be associated with low responders in the vildagliptin group, but high responders in the berberine group (Figure 2B and Table S12), as determined by their median percentage changes of HbA1c, similar to the contrasting responses of *Bacteroides* spp. to the two drugs (Figure 1E). Furthermore, specific baseline features were consistently associated with antidiabetic effects across OADs groups (Figure S9), including associations between higher abundances of *bsh* genes and greater treatment improvements in metformin (HbA1c) and acarbose group (HOMA‐IR, Gu et al. [4]) (Figure 2C), and those between higher abundances of glycerolipid metabolism genes (*gldA*, *DhaK*, and *DhaL*) and greater HbA1c reduction in metformin and vildagliptin groups (Figure 2D). Conversely, increased baseline abundances of species like *Adlercreutzia equolifaciens*, *Eggerthella lenta*, *A. hallii*, *Eubacterium ramulus*, and *Ruminococcus sp*_5_1_39BFAA were associated with diminished antidiabetic effects in the metformin and vildagliptin groups (Figure S9).

These findings highlight the predictive potential of baseline gut microbiota for treatment responses to different OADs. A recent study identified *Bacteroides* spp. as major producers of microbial dipeptidyl peptidase 4. These bacterial host isozymes were demonstrated to effectively metabolize and inactivate the glucagon‐like peptide 1 [8]. In alignment with this discovery, nonresponders in our vildagliptin study exhibited higher baseline abundances of *Bacteroides* spp. compared with the responders. Furthermore, vildagliptin treatment led to a significant reduction in the relative abundances of multiple *Bacteroides* species. These findings collectively underscore the bidirectional relationships between the gut microbiota and OADs, and their roles in modulating drug efficacy.

Although analyzing pre–post multi‐omics data from clinical trials could effectively mitigate the confounding effects of interindividual variations and environmental exposures [2, 17, 18], this study has inherent limitations. First, excluding the metformin study of Wu et al. [5], all other samples exclusively comprised Chinese T2D patients, possibly constraining the generalizability of the findings to broader populations. Second, there was limited concordance in baseline microbial features associated with treatment responses in the two acarbose studies, suggesting that factors such as small sample sizes and geographical disparities might confound feature selection. Further research involving larger and more diverse cohorts is necessary to thoroughly assess the interactions between gut microbiota, antidiabetic medications, and human metabolic health.

## CONCLUSION

In this study, we conducted a comprehensive analysis of 1,045 metagenomic samples and revealed extensive bidirectional interactions between OADs and the gut microbiota in patients with T2D. Our findings revealed both unique and shared OADs‐induced microbial alterations, uncovering complex interactions between acarbose, a high‐fiber diet, and the gut microbiota, and highlighting the intricate relationships between baseline gut microbiota and therapeutic effects impacting on metabolic health.

## AUTHOR CONTRIBUTIONS

Huahui Ren, Huanzi Zhong, Karsten Kristiansen, Yanyun Gu, Weiqing Wang, and Guang Ning designed the study. Fengyi Yuan, Yanyun Gu, and Yifei Zhang conducted the clinical trial, enrolled, and managed the patients. Shujie Wang and Tingting Li were responsible for collecting biological samples and clinical data. Huahui Ren, Zhun Shi, Fangming Yang, Min Li, and Jiahui Zhu carried out bioinformatic analyses. Huahui Ren, Huanzi Zhong, Zhun Shi, Fangming Yang, Kui Wu, and Junhua Li contributed to data interpretation. Huahui Ren, Huanzi Zhong, and Yanyun Gu wrote the manuscript. Karsten Kristiansen revised the manuscript. All authors discussed the results, read and approved the final manuscript.

## CONFLICT OF INTEREST STATEMENT

The authors declare no conflict of interest.

## ETHICS STATEMENT

The study was approved by the ethics committee of Shenzhen People's Hospital and the Institutional Review Board of BGI (BGI‐IRB 17049).

## Supporting information


**Figure S1.** PRISMA flow diagram.
**Figure S2.** Meta‐analysis assessing the effects of different OADs on diabetic parameters.
**Figure S3.** Baseline differences in gut microbial composition across the eight intervention groups receiving only OADs.
**Figure S4.** Changes in gut microbial composition induced by treatment with OADs.
**Figure S5.** Alterations of species‐species networks induced by five oral antidiabetic drugs.
**Figure S6.** Changes in secondary bile acids and associated species induced by acarbose, berberine and glipizide.
**Figure S7.** Longitudinal associations between HbA1c and microbial features.
**Figure S8.** Evaluation of alteration in relative abundance of species induced by acarbose alone and in combination with a high‐fiber diet.
**Figure S9.** Association between baseline microbial features and treatment responses of diabetic parameters.

**Table S1.** PubMed search results of metagenomic studies for the meta‐analysis.
**Table S2.** Phenotypic information and sequence data statistics for 1,045 samples included in this study.
**Table S3.** Comparison of CLR‐transformed relative abundance of species between pre and post‐intervention samples for eight interventional groups.
**Table S4.** Network properties of species co‐occurrence networks in eight intervention groups befroe and after intervention.
**Table S5.** Differential network analysis for species co‐occurrence networks in eight intervention groups before and after intervention.
**Table S6.** Comparison of RPKM of secondary bile acid (SBA) biotransformation genes between pre and post‐intervention samples for eight interventional groups.
**Table S7.** Comparison of relative abundances of 11 microbial genes between pre and post‐intervention samples for eight interventional groups.
**Table S8.** Comparisons of  CLR‐transformed relative abundance of species between pre‐ (D0) and post‐intervention samples (D28, D56, D84) in Acarbose alone and Acarbose+WTP interventional groups.  WTP: a high‐fiber diet composed of whole grains, traditional Chinese medicinal foods, and prebiotics.
**Table S9.** Comparisons of RPKM of SBA biotransformation genes between pre‐intervention (D0) and post‐intervention (D28, D56, D84) in Acarbose alone and Acarbose+WTP interventional groups.
**Table S10.** Associations between baseline microbial features and glycemic response in seven intervention groups (GuYY_2017_Acarbose, GuYY_2017_Glipizide, SZ_2023_Metformin, ZhangXY_2022_Acarbose, ZhangXY_2022_Vlidagliptin, ZhangYF_2020_Berberine, ZhangYF_2020_Placebo) estimated by partial Spearman's rank correlation (adjusted for baseline levels of age, sex, BMI and glycemic characteristics).
**Table S11.**  Response variance in diabetic parameters explained by baseline microbial features in seven intervention groups (GuYY_2017_Acarbose, GuYY_2017_Glipizide, SZ_2023_Metformin, ZhangXY_2022_Acarbose, ZhangXY_2022_Vlidagliptin, ZhangYF_2020_Berberine, ZhangYF_2020_Placebo) using the linear regression method (the Elastic Net was applied for feature selection).
**Table S12.** Comparisons of baseline abundances of Bacteroides spp. between the low responders (LR) and high responders (HR) of HbA1c in vildagliptin and berberine groups using Wilcoxon rank‐sum test and logistic regression (adjusted for baseline levels of age, sex, BMI and HbA1c).

## Data Availability

The data that support the findings of this study are openly available in China Nucleotide Sequence Archive (CNSA) at https://db.cngb.org/search/project/CNP0004692/, reference number CNP0004692. The code has been uploaded to GitHub repository at https://github.com/rusher321/OAD_Microbiota. Supplementary materials (methods, figures, tables, scripts, graphical abstract, slides, videos, Chinese translated version and update materials) may be found in the online DOI or iMeta Science http://www.imeta.science/.
