# Supplementary material for: Deciphering unique and shared interactions between the human gut microbiota and oral antidiabetic drugs
Source: Imeta. 2024 Mar 13;3(2):e179. doi: 10.1002/imt2.179 (PMC11170963; doi:10.1002/imt2.179)
Supplement: Supplementary file 1 — Figure S1. PRISMA flow diagram. Figure S2. Meta‐analysis assessing the effects of different OADs on diabetic parameters. Figure S3. Baseline differences in gut microbial composition across the eight intervention groups receiving only OADs. Figure S4. Changes in gut microbial composition induced by treatment with OADs. Figure S5. Alterations of species‐species networks induced by five oral antidiabetic drugs. Figure S6. Changes in secondary bile acids and associated species induced by acarbose, berberine and glipizide. Figure S7. Longitudinal associations between HbA1c and microbial features. Figure S8. Evaluation of alteration in relative abundance of species induced by acarbose alone and in combination with a high‐fiber diet. Figure S9. Association between baseline microbial features and treatment responses of diabetic parameters. [file IMT2-3-e179-s002.docx]

**Supporting information to**

**Deciphering Unique and Shared interactions between the Human Gut Microbiota and Oral Antidiabetic Drugs**

**Runing Title**: **Interactions between the Human Gut Microbiota and Oral Antidiabetic Drugs**

Huahui Ren^1,2#^, Zhun Shi^1^, Fangming Yang^1^, Shujie Wang^3,4^, Fengyi Yuan^5^, Tingting Li^3,4^, Min Li^1^, Jiahui Zhu^1^, Junhua Li^1,6^, Kui Wu^1,7^, Yifei Zhang^3,4^, Guang Ning^3,4^, Karsten Kristiansen^1,2*^, Weiqing Wang^3,4*^, Yanyun Gu^3,4*^, Huanzi Zhong^1*^

^1^BGI Research, Shenzhen 518083, China.

^2^Laboratory of Genomics and Molecular Biomedicine, Department of Biology, University of Copenhagen, 2100 Copenhagen, Denmark

^3^Department of Endocrine and Metabolic Diseases, Shanghai Institute of Endocrine and Metabolic Diseases, Ruijin Hospital, Shanghai Jiao Tong University School of Medicine, Shanghai 200000, China.

^4^Shanghai National Clinical Research Center for Metabolic Diseases, Key Laboratory for Endocrine and Metabolic Diseases of the National Health Commission of the PR China, Shanghai National Center for Translational Medicine, Ruijin Hospital, Shanghai Jiao Tong University School of Medicine, Shanghai 200000, China.

^5^Department of Endocrinology and Metabolism, Shenzhen People's Hospital, Shenzhen 518083, China.

^6^Shenzhen Key Laboratory of Unknown Pathogen Identification, BGI Research, Shenzhen 518083, China.

^7^Guangdong Provincial Key Laboratory of Human Disease Genomics, Shenzhen Key Laboratory of Genomics, BGI Research, Shenzhen 518083, China

*Correspondence: [zhonghuanzi@genomics.cn](mailto:zhonghuanzi@genomics.cn) (Huanzi Zhong); [guyanyun@sjtu.edu.cn](mailto:guyanyun@sjtu.edu.cn) (Yanyun Gu); [Wqingw61@163.com](mailto:Wqingw61@163.com) (Weiqing Wang); [kk@bio.ku.dk](mailto:kk@bio.ku.dk) (Karsten Kristiansen)

**Methods**

**STUDY PARTICIPANT DETAILS AND METHOD DETAILS**

The GUTT2DM (Effect of Metformin on Gut Microbiota Changes and Glycemic Control of Newly Diagnosed Type 2 Diabetes) study was an open-labelled, single-arm clinical trial (ClinicalTrials.gov number, NCT04426422) designed to investigate the effect of metformin on the gut microbiota and glycemic control in patients with newly diagnosed T2D. Participants were enrolled between March 2017 and Jun 2018 at Shenzhen People’s Hospital, China. The study was approved by the Ethics Committee of the Shenzhen People’s Hospital. All participants provided written informed consent prior to enrollment.

**Participants and intervention procedure.**

Fifty-two eligible participants for the trial were patients with newly diagnosed T2D and were drug naïve for glycemic control. The eligible participants were given an oral metformin monotherapy for 3 months, and patients visited the hospital every month until the end of the study. Forty-seven participants completed the treatment.

**The detailed inclusion criteria.**

Patients were eligible to be included in the study only if they meet all the following criteria:

1. Diagnosed T2D according to the 1999 WHO diagnostic criteria;
2. No history of oral antidiabetic medication or insulin injection;
3. HbA1c levels ≤ 10%;
4. Tested negative for urinary ketones;
5. BMI of ≤ 35 kg/m².

**The detailed exclusion criteria.**

Patients were excluded from the study if they met any of the following criteria:

1. Severe gastrointestinal diseases such as colorectal cancer, irritable bowel syndrome,

inflammatory bowel disease, chronic or acute diarrhea, and long-term constipation;

1. Receiving gastrointestinal or abdominal surgery within the past year;
2. Severe cardiac conditions graded as ≥ 3 according to the New York Heart Association functional classification;
3. Psychiatric disorders, infectious diseases, tumors, or severe anemia;
4. Severe autoimmune diseases;
5. Acute diabetic ketoacidosis (DKA) or hyperosmolar coma within the three months;
6. Known history of allergy to metformin or other serious drug allergies;
7. Receiving antibiotics or corticosteroids in the past three months.

**Biochemical measures**

Fasting and 2-hour postprandial (after a 75 g oral glucose tolerance test) blood samples were collected from the antecubital vein at both the baseline visit and after 3 months of Metformin treatment for each participant. All participants underwent measurements for glycemic measures [fasting plasma glucose (FPG), 2-hour postprandial plasma glucose (PPG120), and HbA1c] and insulin measures [fasting plasma insulin (ins0), 2-hour postprandial plasma insulin (ins120)]. FPG and PPG were measured using the glucose oxidase or hexokinase method. HbA1c was measured within 4 weeks after collection by high-performance liquid chromatography using the VARIANT II Hemoglobin Testing System (Bio-Rad Laboratories, Hercules, CA, USA). Fasting and 2-hour insulin were measured using electrocheuminescence immunoassay “ECLIA” on cobase601 immunoassay analyzer (Roche Diagnostic, Basel, Switzerland).

**STUDY INCLUSION AND PUBLIC DATA COLLECTION**

**Study inclusion and public data collection**

PubMed was used to conduct a search for shotgun metagenomic studies investigating the effects of oral antidiabetic drugs (OADs) on the gut microbiota in treatment-naïve type 2 diabetes (TN-T2D). As search terms we used "(((((shotgun sequencing) AND (human)) AND (metagenomics)) OR (gut microbiota)) AND (type 2 diabetes)) AND (treatment) AND (clinicaltrial [Filter]) AND (in the last 5 years [Filter])" and collected results up to September 18, 2022. A total of five studies meeting the search criteria were included. **Table S1** provides further details regarding the exclusion and inclusion criteria for the studies.

In addition to the PubMed search, we also included a metformin study with Chinese TN-T2D patients conducted as part of this study (**GUTT2DM**), as well as one clinical trial on Chinese T2D patients who received acarbose alone or in combination with a high-fiber diet consisting of a mixture of whole grains, traditional Chinese medicinal foods, and prebiotics WTP) (ChiCTR-TRC-140049959) [19]. The GUTT2DM study (Effect of Metformin on Gut Microbiota Changes and Glycemic Control of Newly Diagnosed Type 2 Diabetes) study was an open-labelled, single-arm clinical trial (ClinicalTrials.gov number, NCT04426422) designed to investigate the effect of metformin on the gut microbiota in patients with newly diagnosed T2D.

The **GUTT2DM** study is registered in ClinicalTrials.gov (Identifier: NCT04426422). This trial was conducted at Shenzhen People’s Hospital between March 2017 and Jun 2018, including 52 newly diagnosed T2D patients receiving a 3-month treatment with metformin. All enrolled participants in this study provided informed consent form before undergoing physical examinations and biomaterial collection. Forty-seven participants completed the treatment. A total of 94 fecal samples were self-collected before and after treatment. The detailed inclusion and exclusion criteria are described in Supplementary Methods. The fecal samples from the GUTT2DM study were self-collected in the hospital before and after 3-month treatment and were stored immediately at –80 °C before DNA extraction. The fecal DNA was extracted as previously described [12], and shotgun metagenomic data were generated using BGISEQ-500 sequencing (single-end; read length of 100 bp).

A total of 1,045 raw FASTQ files were obtained from the six included studies: 1) 188 shotgun metagenomic samples were obtained from Gu et al. [6] (Acarbose and Glipizide); 2) 65 from Wu et al. [16] (Metformin); 3) 172 from Zhao et al. [19] (the GUT2D study, Acarbose); 4) 362 from Zhang et al.2020 [18] (Berberine and Placebo), 5) 164 from Zhang et al. 2022 [17] (the VISA-T2D study Acarbose and Glipizide) and 6) 94 from the current study (the current GUTT2DM study, Metformin). **Table 1** provides a detailed summary of all included studies, including information on the drug used, duration of treatment, drug dosage, and demographical characteristics of the participants.

**METAGENOMIC ANALYSIS**

All of the downloaded metagenomic datasets were processed using the same bioinformatic pipeline. First, low-quality reads were removed using fastp [4] (version 0.20.1) with default parameters. Bowtie2 [8] (version 2.4.2) with default parameters was used to remove human-derived sequences (database hg38) to generate non-human high-quality reads (referred to as clean reads).

Taxonomic profiling of clean reads was performed at the species level using MetaPhlAn2 [14] (version 2.7.0) with default parameters, identifying 1,054 species that were presented in at least 1 fecal sample. Next, we excluded low-prevalence microbial species with a prevalence less than 20% across all samples, resulting in 117 common species for subsequent analysis. Two measures for microbial alpha diversity (Shannon index and richness) and four measures for beta diversity (Bray-Curtis, Hellinger and Spearman dissimilarity, and Jensen-Shannon divergence) at the specie levels were calculated (R, package ‘vegan 2.5-7’, v1.3-1). Bray-Curtis, Hellinger, Spearman and JSD uniqueness for individuals in each group was then calculated using the strategy adopted by Wilmanski *et al* [15].

Functional profiling was performed at the KO levels using HUMAnN2 [5] (version 0.11.1). After excluding low-prevalence (< 20% prevalence) microbial KOs, we focused on 11 KOs associated with microbial metabolites relevant to T2D or metabolic disorders, including those involved in butyrate production: K01034 (AtoD), K00634 (ptb), K00929 (buk); histidine metabolism (imidazole propionate production): K01745 (hutH) and K17363 (urdA); and glycerolipid metabolism: K00005 (gldA), K00864 (gldK), K03621 (plsX), K05878 (DhaK), K05879 (DhaL), K05881 (DhaM). The relative abundances of these KOs were calculated from HUMAnN2. Additionally, we constructed a local protein database by searching the UniProt (Universal Protein Resource) database for available non-redundant microbial proteins involved in secondary bile acid (SBA) biotransformation, including the bile salt hydrolase (*bsh*), 7β-hydroxysteroid dehydrogenase (7β-*hsdh*) and two key BA-inducible operons encoding genes (*baiE* and *baiI*). Clean reads were then annotated using DIAMOND[2] at 60% identity. The RPKM (Reads Per Kilobase per Million mapped reads) of the four SBA transformation genes were calculated by FMAP [7] (v 0.15) based on the annotated reads.

**STATISTICAL METHODS**

**Meta-analysis**

To assess the effects of the five different OADs on host diabetic parameters and gut microbial composition, we applied the random-effects meta-analysis model [1] (R, package ‘meta’, v5.5-0) accounting for unobserved heterogeneity among studies. Two estimators based on inverse variance weights and DerSimonian-Laird methods were included to estimate the between-study variance. Specifically, the overall changes in six diabetic parameters (HbA1c, FPG, PPG, Fins, Pins, and HOMA-IR) and two microbial diversity measures (richness and Shannon index) were estimated. The meta-analysis was conducted on 331 newly diagnosed-T2D (ND-T2D) patients from eight treatment groups with five OADs. Excluded were the 43 T2D patients from the study of Zhao et al.[19], who received prior antidiabetic treatments, and 96 ND-T2D patients from the placebo group of Zhang et al. 2020 [18].

**Evaluation of the changes in gut microbial community induced by different OADs.**

The baseline inter-group microbial variations among different OADs-treatment groups, as well as the treatment-induced microbial variations (pre- vs. post-treatment) within each treatment group were assessed using permutational multivariate analysis of variance (PERMANOVA) (R, package ‘vegan’, v2.5-6, function ‘adonis’, permutations = 999). This analysis was performed at the species level using four dissimilarity measures (Bray-Curtis, Hellinger, Jensen-Shannon, and Spearman). The magnitude of the overall compositional variations was quantified using R-squared. Considering the baseline variations in microbial community, all metagenome-related analyses were performed within each study. Additionally, 172 metagenomic samples of 43 T2D patients from Zhao et al. [19] were integrated with the two acarbose monotherapy groups to provide a comprehensive assessment of the impacts of acarbose alone and acarbose combine with a high-fiber diet on the gut microbiota.

To evaluate the impact of OADs and placebo on individual microbial communities, we employed distance matrix-based individual classification. This included computing the four above-mentioned distance matrices based on relative abundances of all microbial species, followed by a nearest neighbor clustering [3]. Correct classification/assignment was achieved when two samples within a cluster belonged to the same individual. The percentage of correct classification/assignment among all individuals was then calculated within each group to assess the magnitude of drug effects on the gut microbiota at the individual level.

Before comparative analyses of the relative abundances of microbial species and pathways between pre- and post-treatment samples in each OAD group, centered log-ratio transformation (CLR) was applied to deal with compositional bias [13] (R, package ‘robCompositions’, v2.3.1). We then employed the Wilcoxon signed-rank test to analyze the differences between pre- and post-treatment samples in CLR-transformed relative abundance profiles of taxa and pathways, KOs, and RPKM of genes involved in BA transformation. The effect size from the Wilcoxon signed-rank test was calculated to estimate the magnitude of the pre-post difference in microbial variables (R package ‘rcompanion’, version 2.3.25). The Benjamini-Hochberg (BH) method was to use to correct for multiple comparisons at the species and pathway levels. A BH-adjusted *p* value < 0.05 was considered statistically significant.

**Evaluation of the change in microbial co-occurrence network induced by different OADs.**

Microbial co-occurrence networks were built for each treatment group for pre- and post-treatment samples using the CLR-transformed species relative abundance profiles using NetCoMi [11]. The correlation matrices were calculated using Spearman’s rank correlation. Binary-valued adjacency matrixes were generated by converting absolute Spearman’s rho values of 0.5 or higher to 1, indicating the presence of strong correlations, while smaller values were set to 0. The topological propertied of each network, including the number of edges, positive edge percentage, edge density, clustering coefficient, modularity, and connectivity, were calculated using *netConstruct* function (R package ‘*NetCoMi*’, version 1.0.3). For each species, ecological measures including degree, betweenness, closeness, and eigenvector centrality were computed within the network.

Differential network analysis was conducted to compare the pre- and post-treatment networks within each treatment group by examining the difference in centrality measures of the identified central nodes (nodes with a centrality value above the empirical 75th quartile) using the Jaccard index. The degree, betweenness, closeness, and eigenvector centralities were considered. The Jaccard index was used to quantify the similarity between two sets of most central nodes in the pre- and post-treatment networks and ranged from 0 to 1, with larger values indicating a higher degree of similarity. We estimated the statistical significance of the Jaccard index by comparing the observed values with those expected under random conditions. For each drug group, the differential edges that showed significant differences between pre- and post-treatment networks were identified using a permutation test, with a significance threshold of *p* < 0.005 based on 1000 permutation.

**Evaluation of the changes in bile acid metabolism induced by different OADs.**

Wilcoxon signed-rank test was conducted to assess the changes in RPKM of genes involved in BA biotransformation, and the relative abundances of the 11 KOs involved in butyrate production, histidine metabolism, and glycerolipid metabolism induced by the OADs and placebo. The relative levels of BAs, including ursodeoxycholic acid (UDCA) and deoxycholic acid (DCA), were only measured and analyzed in Gu et al. 2017 and Zhang et al. 2020. MaAsLin2 [9] was performed to evaluate the associations between BA genes and host BA levels, as well as the associations between BA genes and microbial species on baseline samples from these two studies (R, package ‘Maaslin2’, version 1.8.0). A BH-adjusted *p* value < 0.05 was considered significant.

**Longitudinal association analyses between microbial variables and glycemic parameters**.

A generalized estimated equations (GEE) model was built to assess longitudinal associations between changes in CLR-transformed abundance of microbial features (including 45 species and 10 functional genes that exhibited consistent changes in relative abundance in at least two different drug treatment arms, Figure S4c) and levels of HbA1c in seven OAD-treatment groups, after adjustment for age, sex, and BMI (R, package ‘geeM’, v0.10.1) [10]. The GEE model for individual species/genes and clinical parameter is shown below:

$${Species/Gene} \sim\beta1 HbA1c+ \beta2 sex+\beta3 age +\beta4 BMI+ \epsilon$$

Where $\beta1, \beta2, \beta3, and \beta4$ indicates the individual regression coefficient of HbA1c, sex, age, and BMI, respectively; and $\epsilon$ indicates the error term. The Wald statistic and *p* value of the regression coefficient of HbA1c ($\beta1$) were calculated to measure the significance and direction of its associations with microbial features. A BH-adjusted *p* value < 0.05 was considered statistically significant.

**Evaluation of the association between baseline microbial variables and OAD-related treatment responses**.

To assess the association between baseline microbial variables and OADs-related treatment responses, we employed machine learning-based Elastic Net regression [20] (R, package ‘glmnet’, version 4.1.4). Here, we excluded the metformin group in the study of Wu et al. 2017 due to its relatively small sample size and used the remaining six treatment groups and one placebo group for analysis. We normalized the changes in post-treatment diabetic parameters to the baseline values as follows:

For each individual, percentage change (PC) from baseline of a given parameter was calculated using the following equation:

${Percentage change}_{GI}$= $\frac{{Post\_treatment}_{GI}-{Baseline}_{GI}}{{Baseline}_{GI}}$ * 100%

Where “Baseline” and “Post_treatment” were measured values of the diabetes parameters (HbA1c, FPG, PPG, Fins, Pins, HOMA-IR) from the same individual before and after treatment, respectively. For each treatment group, the PC values from baseline for each variable were considered as the treatment response.

All of the 150 baseline microbial variables, comprising 135 species, 11 KOs, and 4 BA genes, were included to associate with PCs of diabetes parameters in each treatment group using partial Spearman’s correlation, with adjustment for age, sex, BMI, and the baseline levels of the corresponding diabetic parameter. All of the microbial features that were significantly associated with a specific parameter at a *p* value < 0.05 and an absolute correlation coefficient > 0.3 were included in the Elastic Net model. These microbial features were further selected using Elastic Net with a lambda value that gave a minimum mean error from a leave-one-out cross-validation. This method was applied to control for overfitting and to provide a conservative estimate of model performance. Finally, the microbial features chosen by the Elastic Net were incorporated into the linear model to estimate the explained variance contributed by different factors, and the resulting adjusted R-squared and *p* value from F-test for each factor were recorded.

Association between relative abundances of *Bacteroides* spp. and HbA1c responses to vildagliptin and berberine was evaluated using odd ratio (low responders vs. high responders, defined by median of PC of HbA1c), estimated by logistic regression adjusting for age, sex, BMI, and the baseline levels of HbA1c.

**Supplementary Figures**


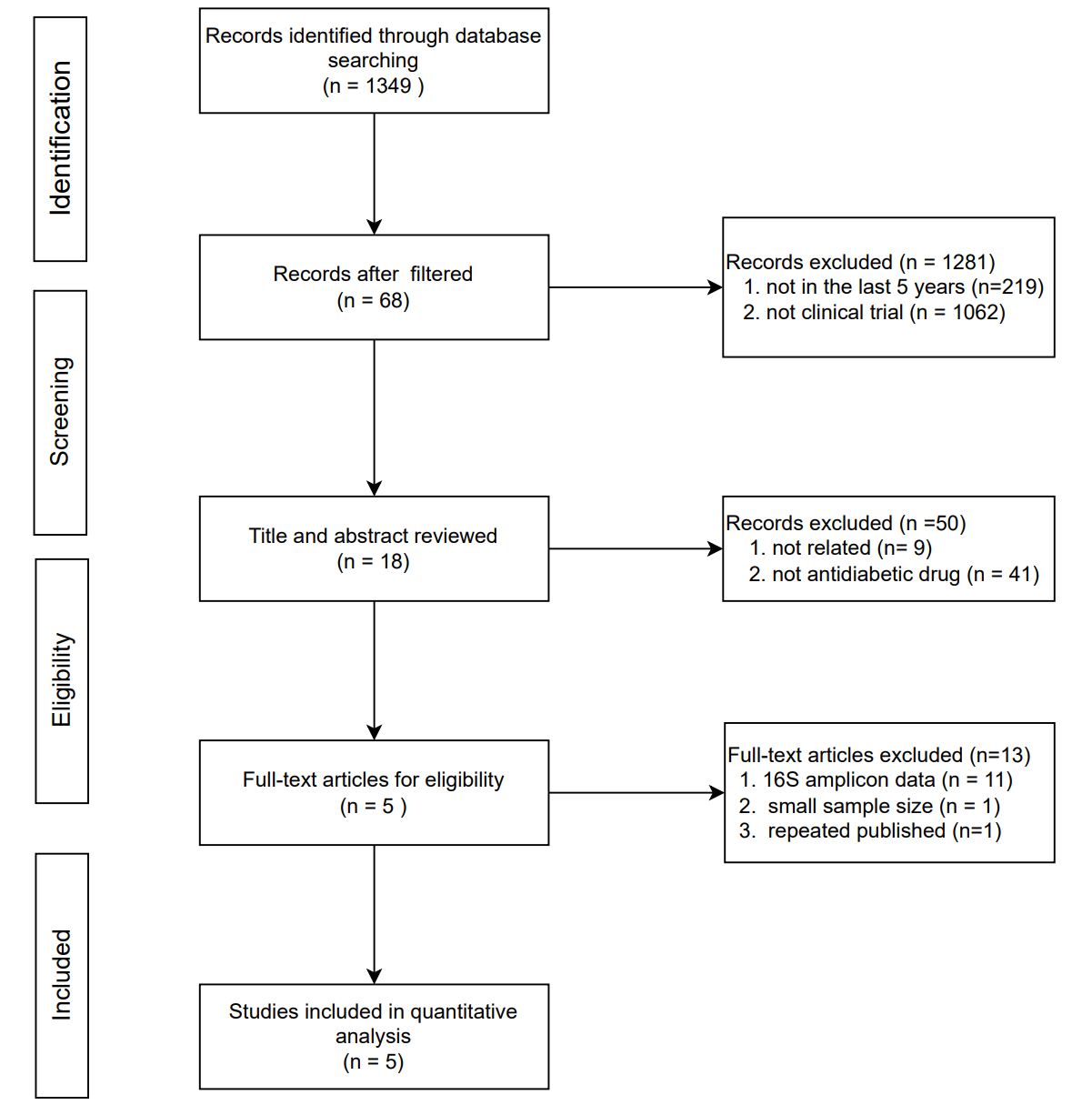


**Figure S1 PRISMA flow diagram.**


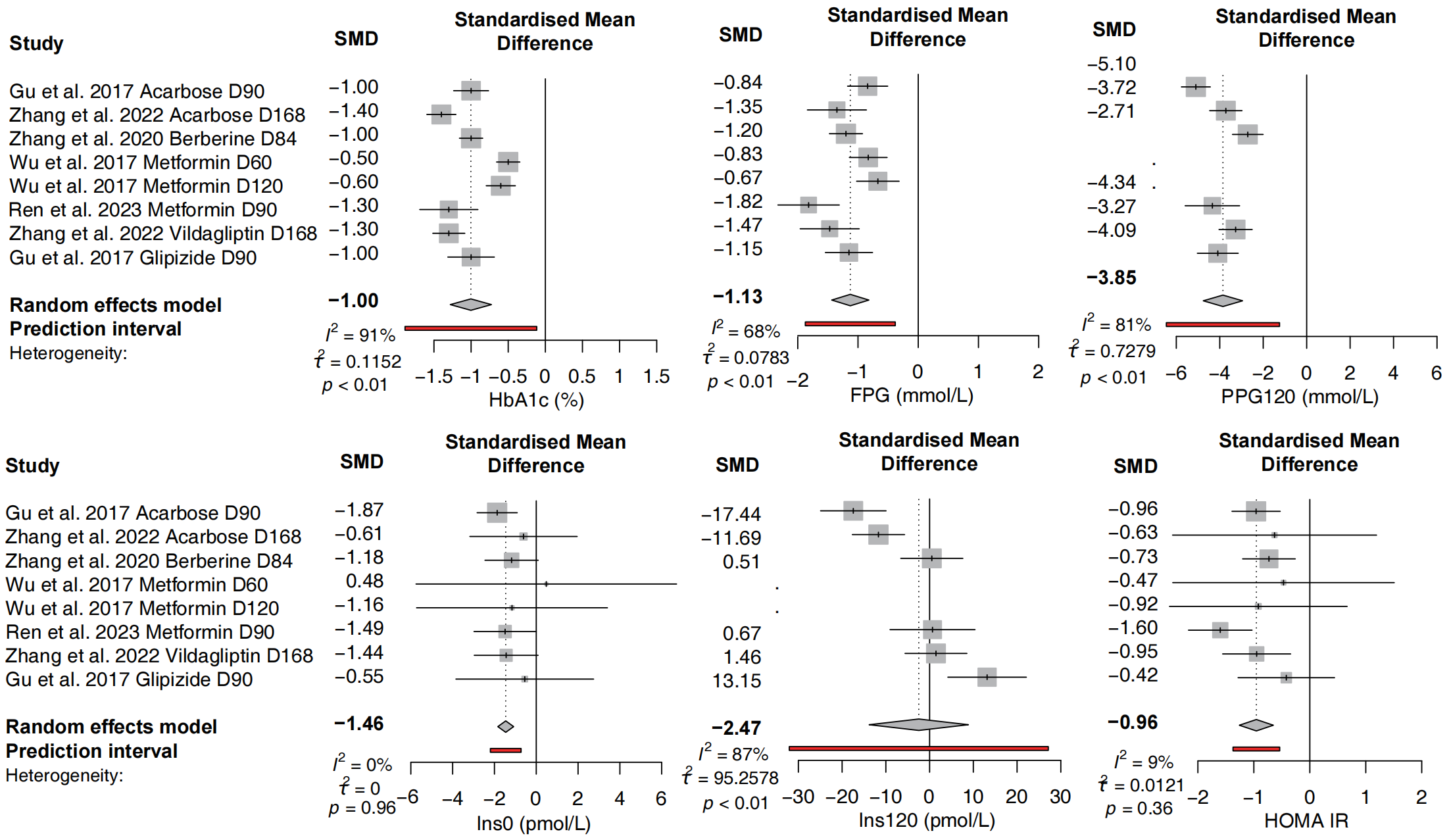


**Figure S2 Meta-analysis assessing the effects of different OADs on diabetic parameters.**

Forest plots showing the mean changes in six diabetic parameters between pre- and post-treatment samples from the five treatment studies and the pooled effect across seven treatment arms (meta-analysis) with 95% CI. A total of 331 patients from seven treatment arms (with two time points D60 and D120 from the Wu et al. 2017 Metformin study) were included for meta-analysis. Detailed information of participants is provided in Table 1.

HbA1c, FPG: Fasting plasma glucose, PPG120: 2-hour postprandial glucose, Ins0: Fasting insulin, Ins120: 2-hour postprandial insulin and HOMA-IR: homeostasis model assessment of insulin resistance.


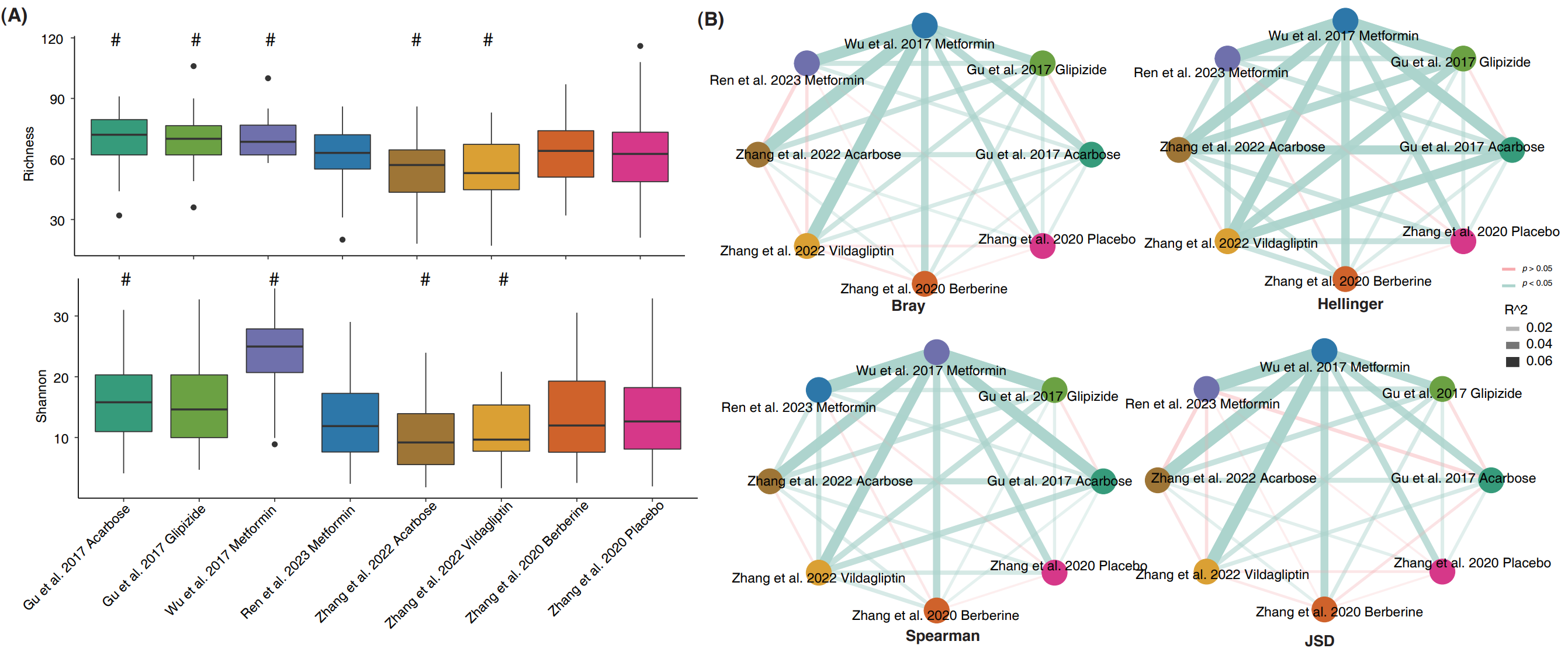


**Figure S3** **Baseline differences in gut microbial composition across the eight intervention groups receiving only OADs.**

**(A)** Boxplots showing the differences in two alpha diversity indices (A: richness, B: Shannon index at species level) among the eight treatment groups at baseline. Wilcoxon rank-sum test for comparisons between a specific group vs. other groups, # indicates *p* < 0.05.

**(B)** PERMANOVA showing the significance and magnitude of baseline microbial variations among the eight groups based on species-level Bray-Curtis dissimilarity, Hellinger distance, Spearman dissimilarity and Jensen-Shannon divergence. Red edges indicate non-significant differences (*p* > 0.05); green edges indicate significant differences (*p* < 0.05), and edge sizes indicate R-squared values.


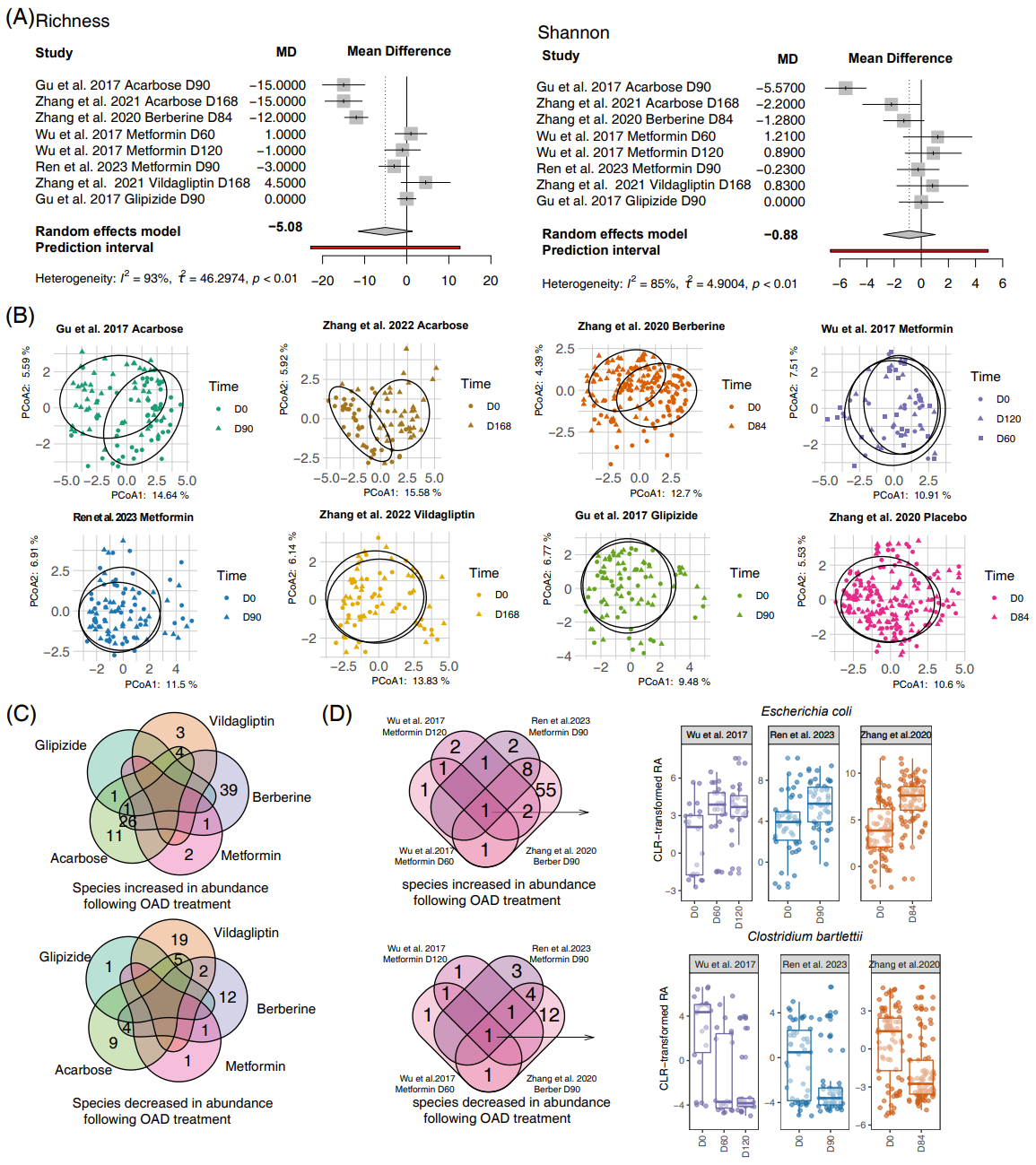


**Figure S4** **Changes in gut microbial composition induced by treatment with OADs.**

(A) Forest plots showing the mean changes in alpha diversity (Richness and Shannon index) between pre- and post-treatment samples from the five treatment studies and the pooled effect across seven treatment groups (meta-analysis) with 95% CI.

(B) Principal coordinates analysis (PCoA) plot illustrating overall changes in gut microbial composition after treatments with acarbose, berberine, metformin, vildagliptin, glipizide, or placebo, using Bray–Curtis dissimilarities at the species level.

(C) Venn plots showing the number of gut microbial species exhibiting shared or specific changes in abundances, increased (left) or decreased (right) across different OADs groups (acarbose, metformin, berberine, vildagliptin, and glipizide). Considering the relatively small sample size in several groups, we defined changes in relative abundance in species based on a *p* value < 0.05 using Wilcoxon signed-rank test.

(D) Venn plots showing the number of gut microbial species exhibiting shared or specific changes in relative abundance in response to treatment with metformin and berberine, increased (top) or decreased (bottom). Boxplots showing a comparison of *Escherichia coli* and *Clostridium bartlettii* between pre- and post-treatment samples in the Wu et al. 2017 study (metformin), our current metformin study, and the Zhang et al. 2020 study (berberine). Significant alteration in relative abundance was determined using *p* < 0.05.


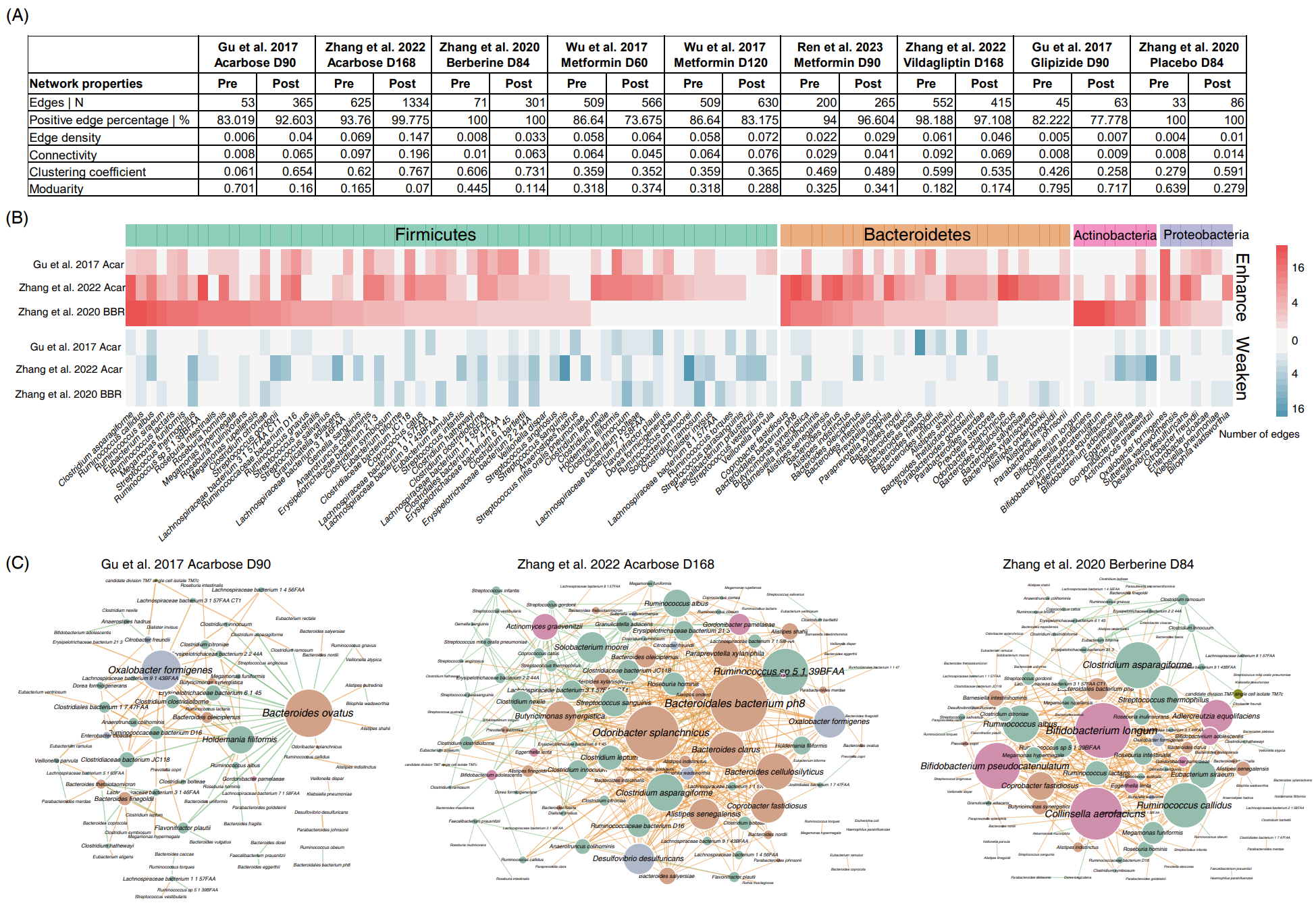


**Figure S5** **Alterations of species-species networks induced by five oral antidiabetic drugs.**

**(A**) Network properties of species-species co-occurrence networks at pre- and post-treatment status for each intervention group. The presence of edges (species-species connections) was defined using an absolute value of Spearman’s rank correlation coefficient greater than 0.5.

**(B**) Heatmap showing the number of enhanced (red, significantly increased absolute values of coefficients) and weakened (blue, significantly decreased absolute values) edges in the differential species-species networks induced by acarbose and berberine treatments. The significance of treatment-enhanced or weakened edges was defined using a *p* < 0.005, estimated by permutation tests (n=1,000) in the NetCoMi package.

**(C**) Differential networks showing the alterations in species-species networks after treatments with acarbose or berberine. Each node represents a species, color-coded by phylum: green for Firmicutes; orange Bacteroidetes; purple Actinobacteria; and blue for Proteobacteria. Solid lines indicate differential edges between pre- and post-treatment networks (green for weakened edges and orange for enhanced edges using a *p* < 0.005 (permutation tests, n=1,000).


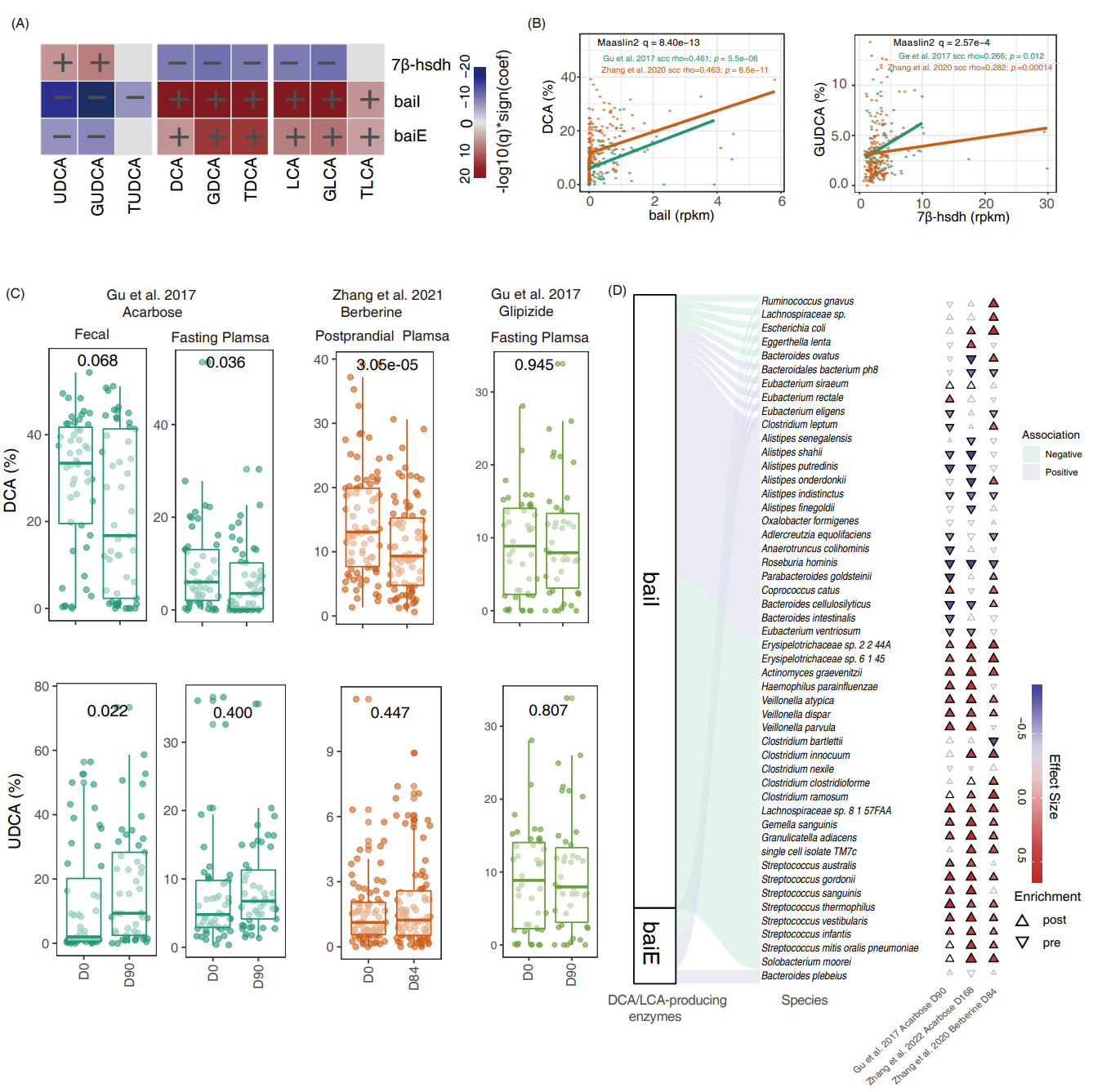


**Figure S6** **Changes in secondary bile acids and associated species induced by acarbose, berberine and glipizide.**

**(A)** Heatmap showing the magnitude of associations between relative abundances of genes involved in microbial SBA biotransformation and relative levels of plasma bile acids in 260 combined baseline samples from the Gu et al. 2017 (n=94) and Zhang et al. 2020 (n=166) studies. DCA, deoxycholic acid; GDCA, glycodeoxycholic acid; GLCA, glycolithocholic acid; GUDCA, glycoursodeoxycholic acid; LCA, lithocholic acid; TDCA, taurodeoxycholic acid; TLCA, taurolithocholic acid; TUDCA, tauroursodeoxycholic acid; UDCA, ursodeoxycholic acid. BH-adjusted *p* value (q) and coefficient (coef) were generated by MaAsLin2. “+” indicates a BH-adjusted *p* value < 0.05 and a coefficient > 0; “-” indicates a BH-adjusted *p* value < 0.05 and a coefficient < 0. Colors are ranked by -log10(q)*sign(coef))

**(B)** Scatter plots showing the associations between the relative abundances of the *baiI* gene and plasma DCA levels, relative abundances of the 7β-*hsdh* gene and plasma GUDCA levels of baseline samples in Gu et al. 2017 and Zhang et al. 2020 studies, respectively. The association *p* values for the combined baseline samples from the two studies were estimated using MaAsLin2. The association *p* values for each study were estimated using Spearman’s rank correlation (SCC).

**(C**) Comparisons of fecal and plasma levels of DCA and UDCA between pre- and post-treatment samples after treatment with acarbose, berberine or glipizide. *P* values were determined using Wilcoxon signed-rank test.

**(D)** Sankey diagram (left) showing species significantly correlated with *baiI* and/or *baiE* (light blue: negative correlations, light purple: positive correlations) in 260 baseline samples from the three groups using MaAsLin2 (Methods). Dot plot (right) showing the changes in relative abundances of *baiI/baiE*-associated species after acarbose or berberine treatments. Effect sizes were calculated using Wilcoxon signed-rank test, blue triangles indicate significantly decreased species and red triangles indicate significantly increased species after treatment. A Benjamini-Hochberg (BH) adjusted *p* < 0.05 was considered statistically significant.


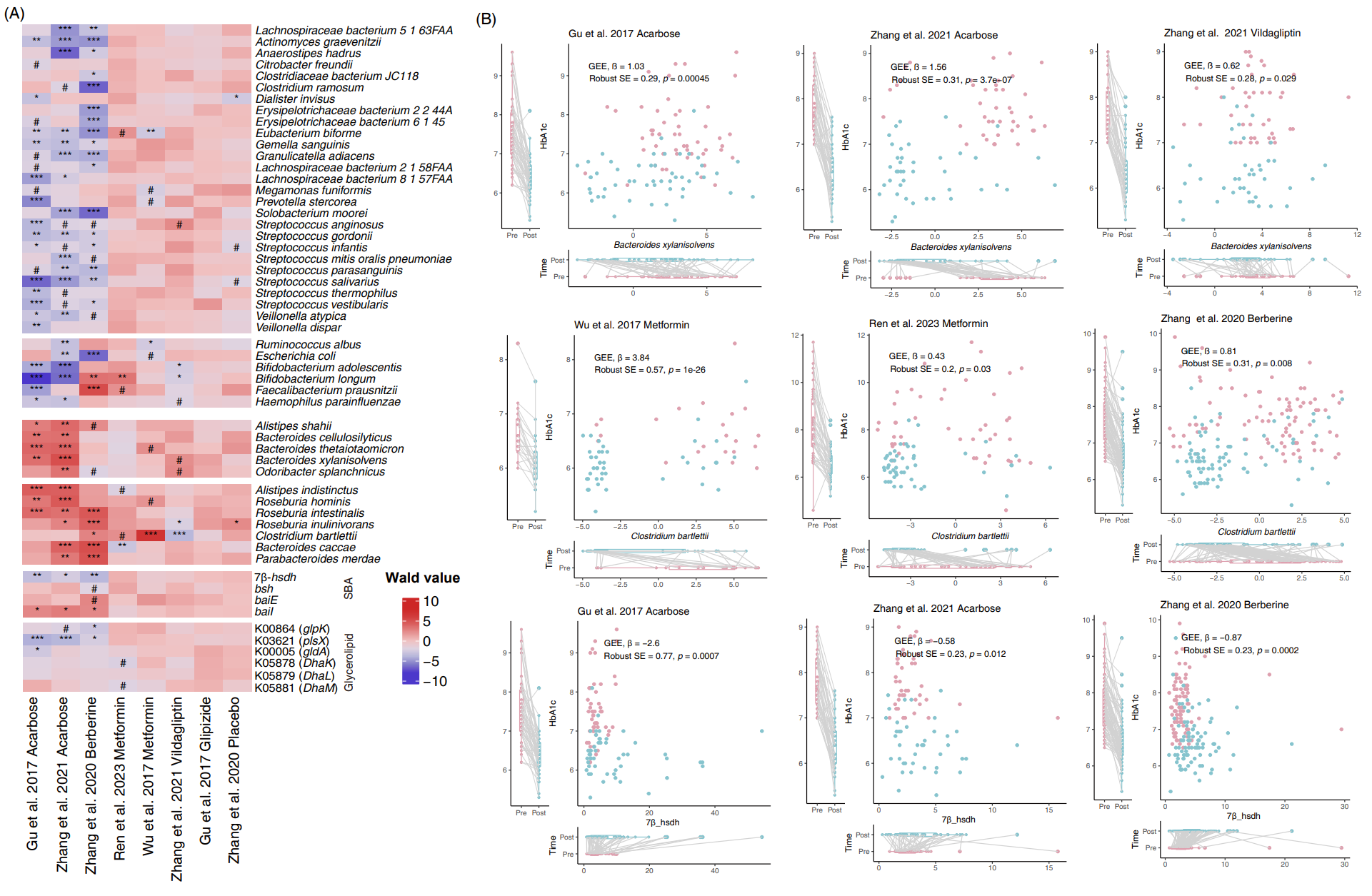


**Figure S7** **Longitudinal associations between HbA1c and microbial features.**

**(A)** Heatmap resulting from Wald statistics of the associations between changes of HbA1c and the species and microbial genes in response to different OADs treatment. This analysis included 45 commonly responding species (defined as those displaying consistent changes in at least two treatment groups, **Figure S4c**), the 10 functional genes involved in secondary bile acid (SBA) biotransformation or glycerolipids metabolism. Wald statistics was calculated based on multivariate regression models using a generalized estimating equations (GEE) approach, adjusting for sex and age. GEE, # *p* < 0.05; * BH-adjusted *p* < 0.05; **, BH-adjusted *p* < 0.01；***, BH-adjusted *p* < 0.001. Microbial genes involved in SBA biotransformation: *7β-hsdh*, *baiI*, *baiE*, and *bsh*; microbial genes involved in glycerolipid metabolism: K05878 (DhaK), K05979 (DhaL), K05881 (DhaM), K00005 (gldA), K00864 (glpK), and K03621 (plsX). Blue indicates negative longitudinal association between changes in species/genes and HbA1c. Red indicates positive longitudinal association between changes in species/genes and HbA1c.

**(B)** Scatter plot of the individual levels of HbA1c against the relative abundances of *B.* *xylanisolvens* (up panel)*, C. bartlettii* (middle panel) and *7β-hsdh* (bottom panel) pre- and post-treatment. Coefficient value (β) and *p* value for longitudinal associations were calculated by GEE model. Tukey-style box plots showing the levels of HbA1c and centered log-ratio (CLR)-transformed abundances of *B.* *xylanisolvens, C. bartlettii and 7β-hsdh* (at the bottom of the scatter plot) in the corresponding subjects, respectively.


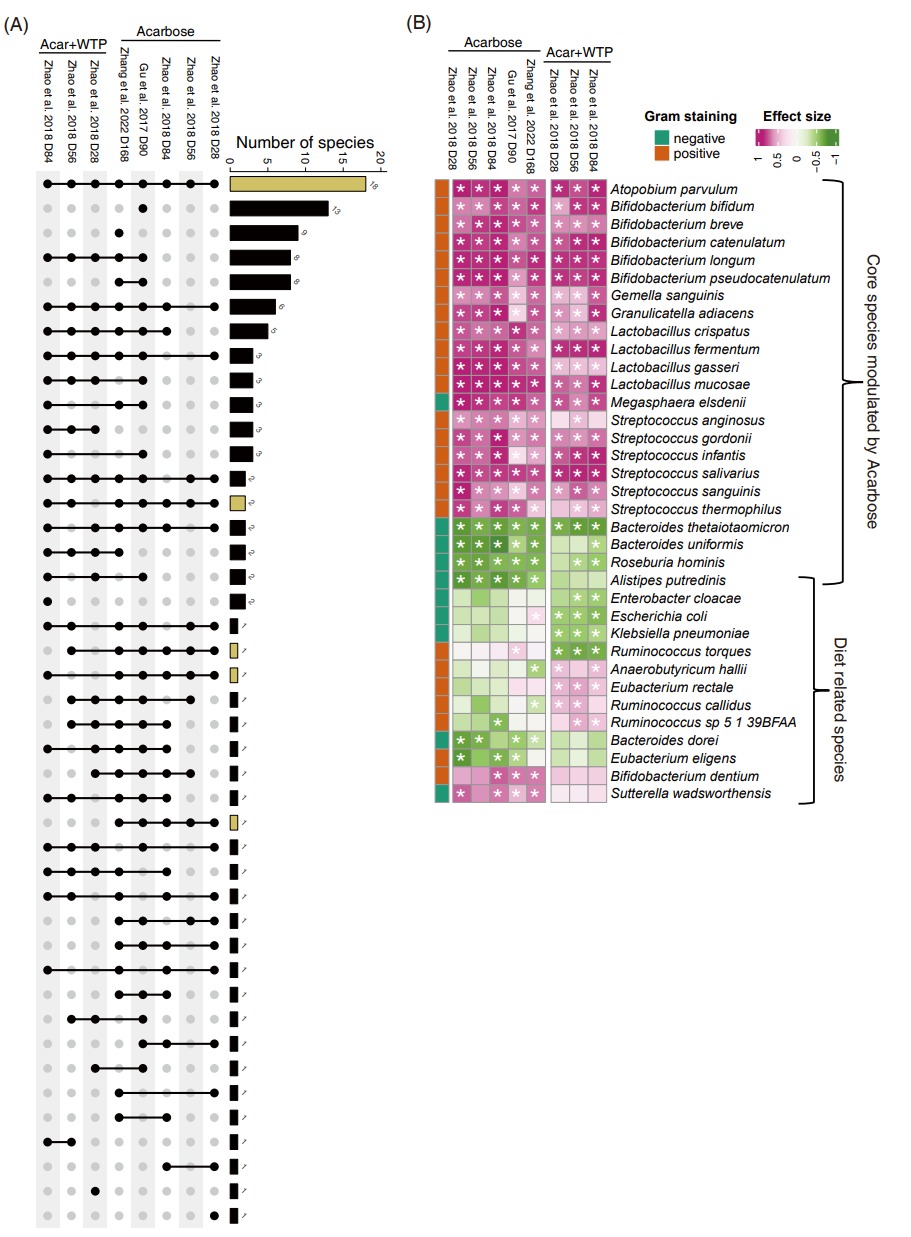


**Figure S8** **Evaluation of alteration in relative abundance of species induced by acarbose alone and in combination with a high-fiber diet.**

**(A)** Upset plot representing the intersection size between species exhibiting significant changes in relative abundance in response to acarbose alone or acarbose combined with a high-fiber diet (Acar+WTP) treatment. Yellow bars indicate the core species exhibiting consistent changes in relative abundance on Day-28, Day-56, Day-84, Day-90, Day-168 of acarbose treatment. Wilcoxon signed-rank tests. A Benjamini-Hochberg (BH) adjusted *p* < 0.05 was considered statistically significant.

**(B**) Heatmap showing core species whose relative abundance was modulated by acarbose alone or acarbose combined with a high-fiber diet (Acar+WTP). Wilcoxon signed-rank tests. *: BH-adjusted *p* < 0.05. “Core species modulated by Acarbose” were defined as those showing consistent alteration patterns across all post-treatment time points with acarbose alone. “Diet related species” were defined as those showing different alteration patterns between acarbose alone and the Acar+WTP group.


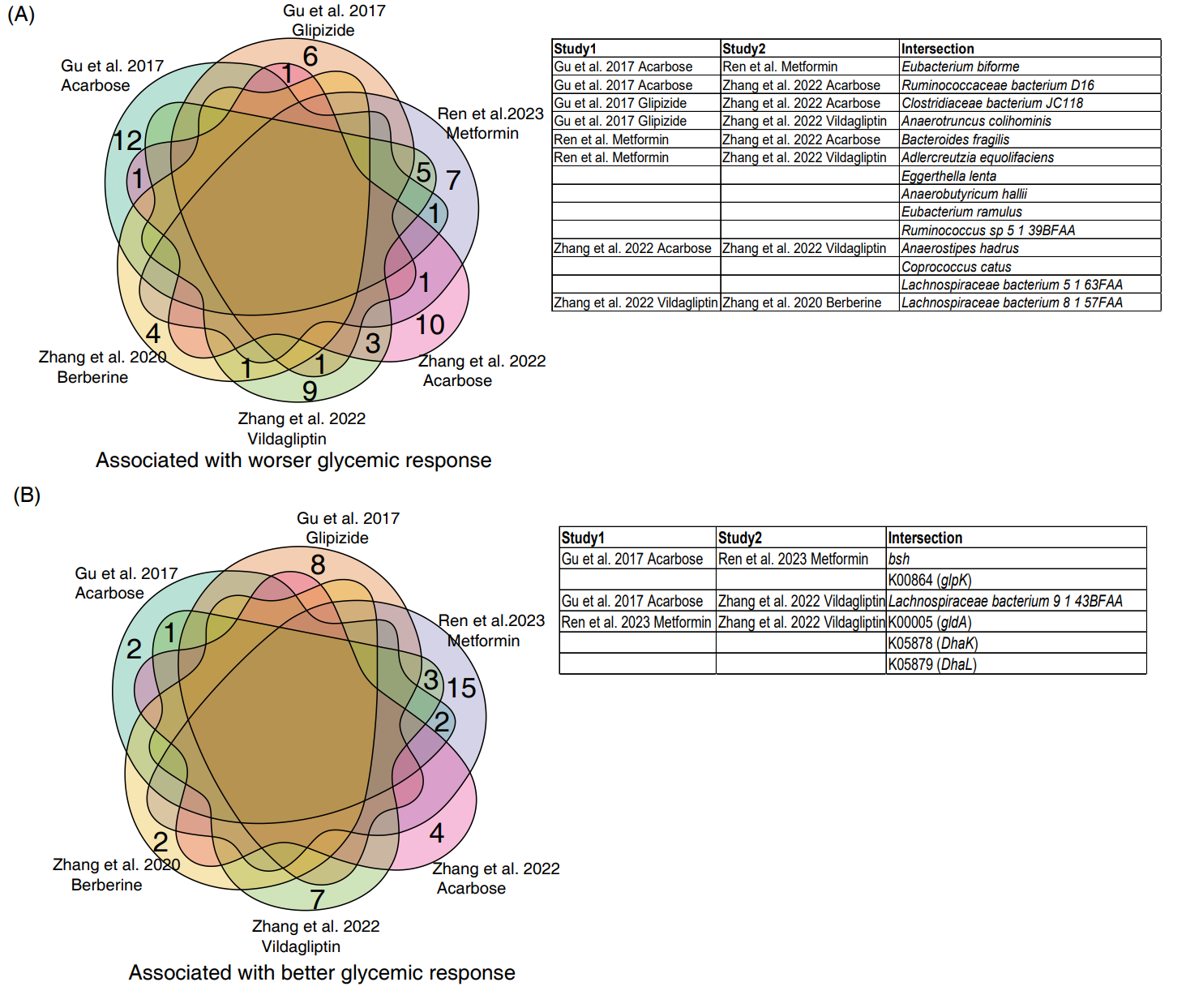


**Figure S9** **Association between baseline microbial features and treatment responses of diabetic parameters.**

**(A-B)**: Venn plots showing the number of gut microbial species exhibiting shared or specific association with a worsened glycemic response (**A**) or an improved glycemic response (**B**) across different OADs groups (acarbose, metformin, berberine, vildagliptin, and glipizide). Treatment responses of diabetic parameters were evaluated using the percentage change from baseline values. Only HbA1c, fasting plasma glucose, and HOMA-IR were considered. Significant associations were defined using an absolute correlation coefficient > 0.3 and *p* value < 0.05.

**Reference**

1. Borenstein, Michael, Larry V. Hedges, Julian P.T. Higgins, and Hannah R. Rothstein. 2010. “A Basic Introduction to Fixed-Effect and Random-Effects Models for Meta-Analysis.” *Research Synthesis Methods* 1: 97–111. https://doi.org/10.1002/JRSM.12.

2. Buchfink, Benjamin, Klaus Reuter, and Hajk Georg Drost. 2021. “Sensitive Protein Alignments at Tree-of-Life Scale Using DIAMOND.” *Nature Methods* 18: 366–8. https://doi.org/10.1038/s41592-021-01101-x.

3. Chen, Lianmin, Daoming Wang, Sanzhima Garmaeva, Alexander Kurilshikov, Arnau Vich Vila, Ranko Gacesa, Trishla Sinha, et al. 2021. “The Long-Term Genetic Stability and Individual Specificity of the Human Gut Microbiome.” *Cell* 184: 2302-2315.e12. https://doi.org/10.1016/J.CELL.2021.03.024.

4. Chen, Shifu, Yanqing Zhou, Yaru Chen, and Jia Gu. 2018. “Fastp: An Ultra-Fast All-in-One FASTQ Preprocessor.” *Bioinformatics (Oxford, England)* 34: i884–90. https://doi.org/10.1093/BIOINFORMATICS/BTY560.

5. Franzosa, Eric A., Lauren J. McIver, Gholamali Rahnavard, Luke R. Thompson, Melanie Schirmer, George Weingart, Karen Schwarzberg Lipson, et al. 2018. “Species-Level Functional Profiling of Metagenomes and Metatranscriptomes.” *Nature Methods* 15: 962. https://doi.org/10.1038/S41592-018-0176-Y.

6. Gu, Yanyun, Xiaokai Wang, Junhua Li, Yifei Zhang, Huanzi Zhong, Ruixin Liu, Dongya Zhang, et al. 2017. “Analyses of Gut Microbiota and Plasma Bile Acids Enable Stratification of Patients for Antidiabetic Treatment.” *Nature Communications* 8: 1785. https://doi.org/10.1038/S41467-017-01682-2.

7. Kim, Jiwoong, Min Soo Kim, Andrew Y. Koh, Yang Xie, and Xiaowei Zhan. 2016. “FMAP: Functional Mapping and Analysis Pipeline for Metagenomics and Metatranscriptomics Studies.” *BMC Bioinformatics* 17: 1–8. https://doi.org/10.1186/S12859-016-1278-0/FIGURES/6.

8. Langmead, Ben, and Steven L. Salzberg. 2012. “Fast Gapped-Read Alignment with Bowtie 2.” *Nature Methods* 9: 357–359. https://doi.org/10.1038/nmeth.1923.

9. Mallick, Himel, Ali Rahnavard, Lauren J. McIver, Siyuan Ma, Yancong Zhang, Long H. Nguyen, Timothy L. Tickle, et al. 2021. “Multivariable Association Discovery in Population-Scale Meta-Omics Studies.” *PLOS Computational Biology* 17: e1009442. https://doi.org/10.1371/JOURNAL.PCBI.1009442.

10. McDaniel, Lee S, Nicholas C Henderson, and Paul J Rathouz. 2013. “Fast Pure R Implementation of GEE: Application of the Matrix Package.” *The R Journal* 5: 181–187.

11. Peschel, Stefanie, Christian L. Müller, Erika Von Mutius, Anne Laure Boulesteix, and Martin Depner. 2021. “NetCoMi: Network Construction and Comparison for Microbiome Data in R.” *Briefings in Bioinformatics* 22: 1–18. https://doi.org/10.1093/BIB/BBAA290.

12. Qin, Junjie, Yingrui Li, Zhiming Cai, Shenghui Li, Jianfeng Zhu, Fan Zhang, Suisha Liang, et al. 2012. “A Metagenome-Wide Association Study of Gut Microbiota in Type 2 Diabetes.” *Nature* 490: 55–60. https://doi.org/10.1038/nature11450.

13. Quinn, Thomas P., Ionas Erb, Greg Gloor, Cedric Notredame, Mark F. Richardson, and Tamsyn M. Crowley. 2019. “A Field Guide for the Compositional Analysis of Any-Omics Data.” *GigaScience* 8: 1–14. https://doi.org/10.1093/GIGASCIENCE/GIZ107.

14. Truong, Duy Tin, Eric A. Franzosa, Timothy L. Tickle, Matthias Scholz, George Weingart, Edoardo Pasolli, Adrian Tett, Curtis Huttenhower, and Nicola Segata. 2015. “MetaPhlAn2 for Enhanced Metagenomic Taxonomic Profiling.” *Nature Methods* 12: 902–903. https://doi.org/10.1038/nmeth.3589.

15. Wilmanski, Tomasz, Christian Diener, Noa Rappaport, Sushmita Patwardhan, Jack Wiedrick, Jodi Lapidus, John C. Earls, et al. 2021. “Gut Microbiome Pattern Reflects Healthy Ageing and Predicts Survival in Humans.” *Nature Metabolism* 3: 274–86. https://doi.org/10.1038/s42255-021-00348-0.

16. Wu, Hao, Eduardo Esteve, Valentina Tremaroli, Muhammad Tanweer Khan, Robert Caesar, Louise Mannerås-Holm, Marcus Ståhlman, et al. 2017. “Metformin Alters the Gut Microbiome of Individuals with Treatment-Naive Type 2 Diabetes, Contributing to the Therapeutic Effects of the Drug.” *Nature Medicine* 23: 850–8. https://doi.org/10.1038/NM.4345.

17. Zhang, Xiuying, Huahui Ren, Cuiling Zhao, Zhun Shi, Li Qiu, Fangming Yang, Xianghai Zhou, et al. 2022. “Metagenomic Analysis Reveals Crosstalk between Gut Microbiota and Glucose-Lowering Drugs Targeting the Gastrointestinal Tract in Chinese Patients with Type 2 Diabetes: A 6 Month, Two-Arm Randomised Trial.” *Diabetologia* 65: 1613–26. https://doi.org/10.1007/S00125-022-05768-5.

18. Zhang, Yifei, Yanyun Gu, Huahui Ren, Shujie Wang, Huanzi Zhong, Xinjie Zhao, Jing Ma, et al. 2020. “Gut Microbiome-Related Effects of Berberine and Probiotics on Type 2 Diabetes (the PREMOTE Study).” *Nature Communications* 11: 5015. https://doi.org/10.1038/S41467-020-18414-8.

19. Zhao, Liping, Feng Zhang, Xiaoying Ding, Guojun Wu, Yan Y. Lam, Xuejiao Wang, Huaqing Fu, et al. 2018. “Gut Bacteria Selectively Promoted by Dietary Fibers Alleviate Type 2 Diabetes.” *Science* 359: 1151–1156. https://doi.org/10.1126/SCIENCE.AAO5774.

20. Zou, Hui, and Trevor Hastie. 2005. “Regularization and Variable Selection via the Elastic Net.” *Journal of the Royal Statistical Society: Series B (Statistical Methodology)* 67: 301–320. https://doi.org/10.1111/J.1467-9868.2005.00503.X.
